# Analysis of Quality of Nuchal Translucency Measurements: Its Role in Prenatal Diagnosis

**DOI:** 10.1100/2012/482832

**Published:** 2011-12-12

**Authors:** Carmen Comas Gabriel, M. Echevarria, I. Rodríguez, B. Serra

**Affiliations:** Fetal Medicine Unit, Department of Obstetrics and Gynecology, Institut Universitari Dexeus, Gran Vía Carles III 71-75, 08028 Barcelona, Spain

## Abstract

*Objective*. Quantitative analysis of the quality of nuchal translucency (NT) measurements. *Methods*. First-trimester combined screening for Down syndrome was performed to all pregnant women attended in our Department from October 2003 to November 2009. NT was measured according to the Fetal Medicine Foundation (FMF) criteria by 20 trained obstetricians. The performance of NT measurements was retrospectively analyzed with regard to several quality control standards. Accuracy according to experience, professional profile, crown rump length (CRL) values, and FMF certification was statistically tested. *Results*. A total of 14978 NT measurements were assessed. (1) The mean operator-specific median NT-MoM values was 0,98. (2) Mean percentage of cases >95th and <5th centiles were 5,0% and 4,2%, respectively. (3) Logarithmic mean and SD of the NT MoM values were 0,00 and 0,13, respectively. (4) The DR for trisomy 21 at screening time was 90,7% for a FPR of 6,7% for standard screening strategy. (5) According to Cumulative SUM (CUSUM) figures, the performance was more acceptable in FMF-certified operators. *Conclusion*. Overall, quality standards show optimal NT measurements in our unit. Operator experience, a dedicated profile to fetal medicine, CRL over 60 mm, and FMF certification have a significant positive impact on the quality standards.

## 1. Introduction

In the medical field and more precisely in prenatal ultrasound, the concept of quality assessment and certification has only recently emerged. In clinical laboratories, all tests are regularly subjected to quality controls to determine their reliability [1]. However, although rigid standardization of laboratory measurements has been traditionally well established [2, 3], clinically measurements such as ultrasound biometries have only recently been object of interest [1, 4–8].

NT measurement has been shown to be a useful marker for Down syndrome in the late first trimester in both, high-risk and low-risk populations, but only when accompanied by targeted training and ongoing quality assessment [7]. A continuous monitoring and scrupulous evaluations of individual performance is likely to improve NT measurement procedure. Although the trisomy 21 detection rate remains a priority, this indicator cannot be used as a reliable marker of quality, given the low prevalence of this condition. Efforts in quality assurance should include more reliable, realistic, and individualized indicators. The aim of this study is to quantitatively assess the quality of NT measurements over a period of 6 years in our fetal medicine unit, testing different models for quality control.

## 2. Methods

This is a retrospective single-centre study started in October 2003, and ended in November, 2009. First trimester combined screening for Down syndrome (DS) was performed to all pregnant women attended in our Department during this period, including maternal age, biochemistry (Pregnancy-Associated Plasma Protein-A and free *β*-human Chorionic Gonadotrophin), and NT. The maternal serum biochemistry was measured using the Kryptor analyzer (Brahms Diagnostica) in a one or two-step strategy between 15 and 85 mm of crown rump length (CRL) measurements. Scans were carried out by 20 trained obstetricians, transvaginally or transabdominally (depending on fetal and maternal conditions). Voluson 730 and E8 (GE Medical Systems, Zipf, Austria) machines with a 5-MHz transabdominal and/or a 8-MHz transvaginal probe were used. NT was measured according to the FMF criteria although in this series, we include NT measurements from 40 to 85 mm of CRL (we include 40 to 44 mm of CRL from cases taken before FMF current consensus). For combined risk calculation, the SBP-software was used, a commercial accredited national software widely used in our geographic area. Cytogenetic study was recommended when combined risk index was higher than 1/270 at screening time. Outcome followup was retrieved from our database, obtaining 96% completed cases in this specified period. For each individual operator and overall, the performance of the NT measurements was analyzed with regard to the follow quality standards: (1) median NT-multiples of the median (MoM) values and 5th and 95th centiles, (2) percentage of cases below and above the 5th and 95th centiles respectively, (3) logarithmic mean and logarithmic standard deviation (SD) of the NT MoM values, (4) performance of the screening test (detection rate-DR- and false positive rate-FPR-), and (5) cumulative SUMs (CUSUM) tests. CUSUMs tests display a sequential monitoring of a cumulative performance measure over time. After each procedure the CUSUM tests whether the process under scrutiny is “in control”, that is, if the process is performing at an acceptable level. For this study, *K* and *h* values were set at 0,25 and ±9,2, respectively, which corresponds to the statistics suggested in the literature. Here, the CUSUM is designed to detect a shift of half the standard deviation and so *K* = 0.25 (*K* = 0.5 *g*, with *g* the number of standard deviations to be detected) and the limits are set at *h* = ±9.2 to optimize the properties of the test [9]. CUSUM graphs include all consecutive NT measurements during a three-month period, excluding values over 3 mm. As a reference for the expected median NT, the Nicolaides formula was used [10]. Operator-specific NT measurement accuracy according to the experience (sequential number of measurements—first 100 scans compared to all scans after the initial 100-), chronological period (2003–2006 versus 2007–2009), professional profile (fetal medicine dedicated or general obstetric profile), CRL values (≤60 versus >60 mm), and FMF certification was statistically tested by Mann-Whitney U (median NT-MoM), ANOVA (logarithmic mean), and random effects ANOVA (SD of the logarithmic NT MoM values). The percentage of cases under and over the 5th and 95th centiles were compared by Pearson's Chi-squared test.

## 3. Results

A total of 14978 NT measurements were reviewed. The mean maternal age was 33 (range 17–45, SD 3,8) years and the mean gestation age at scan was 11 (range 10–13,6) weeks. The population included 32% over 35 years. Down syndrome was identified in 54 pregnancies. Seven out of 20 operators (35%) had a professional profile dedicated to fetal medicine, and 2 of them (10%) were FMF certified at the time of the study. Eight operators remained from 2003 to 2009, which represents a series of 13840 measurements (6615 in the first period and 7225 in the second one). Six operators performed less than 50 NT measurements and were excluded from the analysis. Epidemiological monitoring involved computing five quality measurements, overall and for each operator.

**Figure 1:**
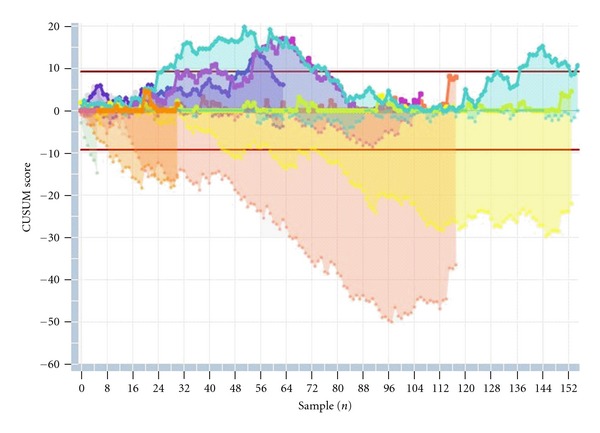
CUSUM graph of consecutive NT measurements for each operator for noncertified operators (during the last three months, excluding measurements >3 mm).

**Figure 2:**
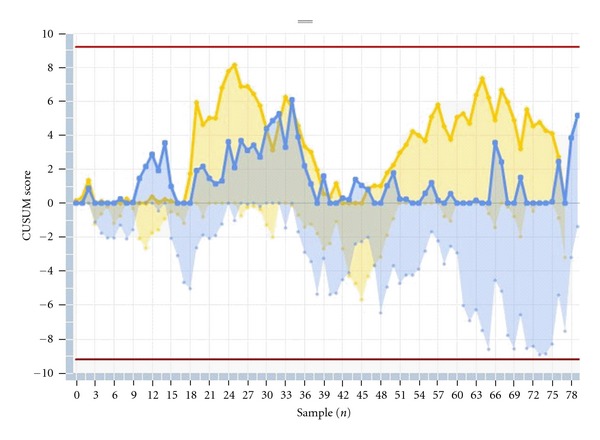
CUSUM graph of consecutive NT measurements for each operator for certified operators (during the last three months, excluding measurements >3 mm).

(1) The mean of all operator-specific median NT-MoM values was 0,98 (targeted value 1,0) (Table 1). Overall, experience (comparing the first and the second chronological period), CRL > 60 mm, and FMF certification had a significant statistical impact improving this standard. FMF-certified operators had a more accurate median NT-MoM (mean of operator-specific medians of 1,00) as compared to the noncertified sonographers (mean of medians of 0,97) (*P* < 0.05). During the study period, the median of NT-MoM of all operators rose significantly, from 0,97 to 0,99 (*P* < 0.05). A professional profile dedicated to US fetal medicine had a tendency to improve the accuracy of measurements although not statistically significant.

(2) Mean percentage of cases over the 95th and below the 5th centiles were 5,0% and 4,2%, respectively (targeted value 5%) (Table 2). Values of CRL < 60 mm and exclusive dedication to fetal medicine had a statistical significant impact improving this standard. 

(3) Logarithmic mean and logarithmic SD of the NT MoM values were 0,00 and 0,13, respectively (mean and SD expected to be 0.00 and 0,08–0,13, resp.) [6] (Table 3). Values of CRL > 60 mm and a dedicated profile had a statistical significant impact improving this standard. Experience and FMF certification had a tendency to reduce the SD (lower dispersion of values) although not statistically significant. 

(4) The DR for DS at screening time was 90,7% for an FPR of 6,7% for standard screening strategy (maternal age, NT, and biochemistry).

(5) Figures 1 and 2 show the CUSUM graph of consecutive NT measurements for each operator, during the last three months, according to FMF certification (excluding measurements >3 mm).  Figure 1 shows the CUSUM graph for the non-FMF-certified operators.  Figure 2 displays the same chart for the FMF-certified operators. 

## 4. Discussion

Increased NT is recognized as a sensitive marker for fetal chromosomal abnormalities. When the karyotype is normal and the NT is enlarged, the fetus is still at increased risk of a broad spectrum of congenital abnormalities, varying from isolated structural defects to genetic syndromes and neurodevelopmental delay [11, 12]. Moreover, recently, it has been demonstrated that many of these cases are linked with submicroscopic chromosomal abnormalities that are typically missed by conventional karyotyping [13]. However, NT screening displays higher variability than biochemical markers due to a lack of automation and significant operator dependence. To minimize variability, international guidelines and quality review programs are being increasingly recommended.

The current cross-sectional study analyzes the performance of NT measurement with regard to several quality standards, individually and for the overall group. Overall, in our series, the quality standards show optimal NT measurements. Moreover, data from our centre are representative of the expected distribution of NT compared to the Nicolaides reference curve. But interestingly, epidemiological monitoring of NT measurements shows that there are differences in each of the quality measures chosen, as previously published [4, 5, 14]. For example, the use of center-specific medians may mask important sonographer-to-sonographer variability. A detailed analysis of the results demonstrates that several operator (experience, dedicated professional profile, and FMF certification) and fetal parameters (range of CRL measurements) have a significant impact on the quality standards. More experienced operators, particularly those with a professional profile focused on fetal medicine, CRL over 60 mm, and FMF certification have a significant positive impact on the quality standards. In our series, typically and as previously published, there was a tendency to move the measurements closer to the median as the experience increases, with lower dispersion of the extreme values [4, 15, 16]. CRL range has also an impact, and measurements of NT in fetuses over 60 mm length seem to be more accurate (in terms of median MoM and logarithmic SD), similar to previous published experiences [4]. Interestingly, each sonographer has the opportunity to compare its own measurements over time with the average measurements performed at the centre. Theoretically, an increase in the variation of NT measurements would lead to suboptimal screening results. Accordingly, we focused on the distribution of these measurements in order to discover systematic differences or changes for the individual examiner and for the total group. Our findings show that when well-trained examiners perform NT screening, continuous evaluation of the distribution of the NT-MoM is a good method to assess the quality of the center and may also be useful to identify individual examiners deviating from the mean performance. Moreover, the CUSUM method has recently received attention in the medical literature owing to its simple formulation and very intuitive representation [9]. This statistical toll graphically presents outcomes of consecutive procedures, estimates the putative factors diminishing the accuracy of the procedure, and assesses the competence of the operator over a certain period of time focusing on systematic and random errors. When applying the CUSUM method, the target, the properties, and the control limits should be prospectively defined, less stringent at the beginning of the learning process, and recalculated according to stricter standards once the initial rates are achieved. In our series, these settings are designed to detect a shift of half the SD (0,125 mm), which corresponds to the statistics suggested in the literature [9]. As shown in the Figures 1 and 2, the performance was more acceptable in FMF-certified operators compared with noncertified operators.  Figure 1 shows the CUSUM graph in the noncertified group, where the CUSUM raises quick alarm in almost all operators. On the contrary, Figure 2 displays the CUSUM chart for the two-certified operators, with all measurements in control during the same three-month period. Besides its relatively simplicity and potential for automation, the main advantages of this method include the early detection of deviations of the measurement compared to other standard quality control indicators. As shown in a recent published study, CUSUM test can be used as a prospective quality control procedure to continuously monitor the performance of sonographers as they assess NT in DS screening [17]. This test model shows close agreement with the retrospective quality review methods on the basis of distribution parameters currently used but with the advantage that it can be prospectively applied, allowing for earlier correction of deviations from target performance. 

In prenatal screening policies, although the highest achievable DR for DS remains a priority, this indicator cannot be used as a reliable marker of quality. The image-scoring methods for quality assessment have been previously introduced in DS quality controls although they have been showed poorly reproducible, too time consuming, and therefore too expensive to apply on a large scale [7, 8]. Extensive qualitative analysis cannot be recommended for ongoing quality control in a NT screening program [5, 8]. These systems might be of more value during the initial training period, or when quantitative assessment indicates the need for further scrutiny. Epidemiological quantitative quality monitoring is a more practical solution. The advantages of quantitative assessment are the relatively simplicity and potential for automation. Moreover, the process of continual assessment and feedback, a well-established principle in business and technology assessments, could be easily adapted to aneuploidy screening in the first trimester. 

Evaluation of prenatal ultrasound measurements for purposes of quality assurance, as known from clinical laboratories analyses [2, 3, 18], has only recently received attention from the fetal medicine community [4, 6, 7, 14, 15, 19, 20]. From a historical perspective, the NT measurement quality certification policy from the FMF (UK) represents the pioneer and more relevant experience in that field [21, 22]. In the United States (US), the Society for Maternal Fetal Medicine created the Nuchal Translucency Quality Review (NTQR) Program to similarly provide education and quality review. Evans has recently demonstrated that a rigid oversight of NT measurements, as practiced by the UK system, is more effective than the currently practiced in the US system [1]. Recent published papers have pointed the effect of deviation of NT measurements on the performance of screening [1, 8, 23, 24]. Recently, Sahota' study has demonstrated significant differences between center and FMF-derived NT MoMs and an increase in NT MoM medians over time. These authors conclude that centers should routinely monitor the quality of NT measurements used to estimate DS screening risk and should provide individualized feedback to sonographers of their measures of central tendency and dispersion to ensure consistent and improved performance. Moreover, NT reference medians adopted from other populations should be assessed and validated against a center's own measurement distribution [20]. Other publications have showed less success for DS screening, probably, this related to the lack of standardization of NT measurements [25, 26]. 

Traditionally, the more effective quality controls have relied on the biochemical screening parameters as compared to ultrasound measurements of NT. Recent publications and our own experience demonstrate that strict quality control of clinically measurements such as NT is possible and as reliable as biochemistry. 

## 5. Conclusion

Our experience in epidemiologic monitoring data shows that the following. (1) Quality standards show optimal NT measurements in our unit. (2) There are differences in each of the quality measures chosen. (3) Several operator and fetal parameters have a significant impact on the quality standards. (4) Despite intense and unified training for all sonographers, NT measurements drift over time for explained and unexplained reasons. Despite appropriate training, experience, accredited certification, and optimal quality standards achieved, continuous monitoring and scrupulous evaluations of individual operators is likely to lead to a better performance of NT screening program. This study demonstrates the importance of ongoing quality assessment. Subsequently, in our centre, regular quality assurance by means of simple and automatic quantitative analysis is going to be conducted at regular 6-month intervals, including prospective CUSUM tool. Commitment to ongoing quality assessment by our scientific community is needed if early screening for DS can maintain high detection rates and low screen-positive rates in clinical practice. 

## Figures and Tables

**Table 1:** Multiples of the median NT values, expressed as median, 5th and 95th centiles, according to the different criteria (operator, period, CRL values, FMF certification, and professional profile).

	MoM-NT			
	Median	5th centile	95th centile	*n*
Operator				
Obs 1	1,00	0,68	1,63	2.364
Obs 2	0,99	0,70	1,45	585
Obs 3	1,13	0,81	1,60	294
Obs 4	1,02	0,64	1,63	905
Obs 5	1,00	0,71	1,40	79
Obs 6	0,83	0,51	1,32	168
Obs 7	0,92	0,58	1,39	51
Obs 8	0,92	0,59	1,42	1.232
Obs 9	0,87	0,41	1,53	114
Obs 10	1,03	0,71	1,68	2.517
Obs 11	0,92	0,60	1,48	2.715
Obs 12	1,04	0,68	1,69	167
Obs 13	1,00	0,64	1,50	1.571
Obs 14	0,95	0,70	1,26	225
Period				
2003–2006	0,97*	0,62	1,67	5560
2007–2009	0,99*	0,67	1,50	6623
CRL (mm)				
≤60	0,96*	0,64	1,61	7.62
>60	1,00*	0,65	1,50	5.467
FMF				
Certified	1,00*	0,67	1,47	1.719
Noncertified	0,97*	0,64	1,58	11.368
Profile				
Dedicated	0,98	0,65	1,56	11.889
Nondedicated	0,97	0,57	1,48	1.198

Overall	0,98	0,64	1,56	13.087

Obs: observer or operator.

US: scans.

FMF: Fetal Medicine Foundation.

*P < 0.05 (comparison between criteria).

**Table 2:** Distribution NT values, expressed in centiles, according to the different criteria (operator, number of consecutive scans, period, CRL values, FMF certification, and professional profile).

	NT				
	≥ 95th centile		≤ 5th centile		Total
	*n*	%	*n*	%	*n*
Operator					
Obs 1	186	6,7**	52	1,9**	2.776
Obs 2	17	2,5**	9	1,3**	669
Obs 3	20	5,7	0	0,0**	352
Obs 4	62	6,3**	38	3,9	987
Obs 5	3	2,1	1	0,7**	141
Obs 6	5	2,5	41	20,6**	199
Obs 7	40	2,8**	92	6,5**	1.407
Obs 8	8	5,1	38	24,4**	156
Obs 9	200	6,7**	43	1,4**	2.968
Obs 10	107	3,6**	190	6,4**	2.969
Obs 11	15	7,0	8	3,8	213
Obs 12	79	4,6	76	4,4	1.712
Obs 13	1	1,8	9	16,4**	55
Obs 14	1	0,4**	8	3,3	243
*n*° US					
First 100 US	63	4,2	117	7,9*	1.486
>100 US	686	5,1	507	3,8*	13.492
Period					
2003–2006	430	6,5*	328	5,0*	6.615
2007–2009	281	3,9*	172	2,4*	7.225
CRL (mm)					
≤ 60	466	5,6*	381	4,6*	8.287
>60	283	4,2*	243	3,6*	6.691
FMF					
Certified	61	3,2*	42	2,2*	1.878
Noncertified	688	5,3*	582	4,4*	13.1
Profile					
Dedicated	691	5,1*	500	3,7*	13.488
Nondedicated	58	3,9*	124	8,3*	1.49

Overall	749	5,0	624	4,2	14.978

Obs: observer or operator.

n° US: number of scans performed.

FMF: Fetal Medicine Foundation.

*P < 0.05 (comparison between criteria).

**P < 0.05 (comparison between obstetricians and expected binomial distribution).

**Table 3:** Logarithmic mean and logarithmic standard deviation (SD) of the NT MoM values, according to the different criteria (operator, period, CRL values, FMF certification, and professional profile).

	log NT		
	mean	SD	*n*
Operator			
Obs 1	0,01	0,12	2.364
Obs 2	0,00	0,10	585
Obs 3	0,05	0,10	294
Obs 4	0,01	0,13	905
Obs 5	0,00	0,09	79
Obs 6	−0,09	0,15	168
Obs 7	−0,04	0,11	51
Obs 8	−0,03	0,13	1.232
Obs 9	−0,07	0,18	114
Obs 10	0,02	0,12	2.517
Obs 11	−0,03	0,13	2.715
Obs 12	0,03	0,13	167
Obs 13	0,00	0,12	1.571
Obs 14	−0,02	0,08	225
Others < 50 US	−0,07	0,16	100

Period			
2003–2006	0,00	0,14	5.56
2007–2009	0,00	0,11	6.623
CRL (mm)*			
≤60	−0,01	0,13	7.62
>60	0,00	0,11	5.467
FMF**			
Certified	0,00	0,11	1.719
Noncertified	−0,01	0,13	11.368
Profile*			
Dedicated	0,00	0,13	11.889
Nondedicated	−0,01	0,14	1.198

Overall	0,00	0,13	13.087

Obs: observer or operator.

US: scans.

FMF: Fetal Medicine Foundation.

*P < 0.05.

**P < 0.01*.*
